# Factors associated with seroprevalence of hepatitis C among dentists at a large Brazilian city

**DOI:** 10.1186/1743-422X-6-228

**Published:** 2009-12-23

**Authors:** Vera Lúcia S Resende, Mauro Henrique G Abreu, Saul M Paiva, Rosângela Teixeira, Isabela A Pordeus

**Affiliations:** 1 Department of Operative Dentistry, Dental School, Federal University of Minas Gerais, Belo Horizonte, Brazil; 2 Department of Community and Preventive Dentistry, Dental School, Federal University of Minas Gerais, Belo Horizonte, Brazil; 3 Department of Paediatric Dentistry and Orthodontics, Dental School, Federal University of Minas Gerais, Belo Horizonte, Brazil; 4 Department of Internal Medicine, Medical School, Federal University of Minas Gerais, Belo Horizonte, Brazil

## Abstract

**Background:**

The aim of the present study was to investigate the seroprevalence and sociodemographic data, health-related and occupational factors and other correlates of sero-posivity among dentists in the city of Belo Horizonte, MG, Brazil.

**Methods:**

A cross-sectional survey was carried out with 1302 dentists in Belo Horizonte, Brazil. All dentists were tested for anti-HCV using a commercially available enzyme-linked immunosorbent assay (ELISA). Individuals positive for anti-HCV were recalled for further evaluation. The presence of HCV RNA in anti-HCV-positive samples was assessed using reverse transcription-polymerase chain reaction (RT-PCR). Data on demographic, behavioural and occupational exposure aspects were collected through questionnaires.

**Results:**

The seroprevalence of anti-HCV was 0.9% (95% IC 0.5-1.7%). The factors associated to the prevalence of hepatitis C were history of blood transfusion (p = 0.002) and having undergone a test for hepatitis C (p = 0.015).

**Conclusions:**

The seroprevalence of anti-HCV among dentists is low. Moreover, no occupational exposure was associated to the seroprevalence of hepatitis C.

## Background

Healthcare-associated infection is an important public health problem worldwide, with ever-increasing interest on the part of politicians, patients and healthcare workers [1,2]. Healthcare providers are at risk of infection from blood-borne pathogens, including hepatitis B (HBV), human deficiency (HIV) and hepatitis C virus (HCV) [3-6]. The transmission of blood-borne viruses in dental offices is a potential hazard to patients and dental staff, particularly to oral and maxillofacial surgeons [7,8]. Chronic hepatitis C is the leading cause of chronic liver disease, cirrhosis, hepatocellular carcinoma (HCC) and liver transplants in Europe and the United States [9-11]. As HCV is transmitted primarily by contaminated blood, it represents a higher risk of nosocomial transmission to patients and healthcare workers [10-12].

Chronic HCV infection is asymptomatic in the majority of infected patients and is not identified unless specific diagnostic tests are performed. Most infected individuals are diagnosed at a later date or when abnormal blood or liver function tests are found in routine examinations for other reasons [12,13].

The current antiviral treatment for chronic HCV infection (pegylated interferon plus ribavirin) provides virus clearance in about 55% of patients with genotype 1 and in 80% of those with genotypes 2 or 3 [14-17]. Hence, the diagnosis of patients infected with chronic HCV is mandatory, since the antiviral treatment might halt or slow the progression of hepatitis to cirrhosis or the development of HCC [13-18].

According to the World Health Organization (WHO) [9], serological HCV tests are strongly recommended for intravenous drug users, people who received plasma-derived products or solid organ transplants before 1992, patients with kidney failure patients on dialysis and children born from women positive for HCV women [19,20] as well as part of the investigation of any liver disease [19,21].

HCV testing is also routinely recommended for healthcare professionals, especially for medical and nursing staffs, following needle stick injuries or mucosal exposure to HCV-positive blood. Dentists appear particularly prone to blood-borne infections, as their routine practice includes the use of sharp instruments in an environment contaminated with saliva and blood. Although virus transmission via saliva may be possible, the major occupational risk is accidental needle stick injuries [22].

Few reports on the prevalence of HCV prevalence in Brazil have been published, with findings ranging from 1.42% in the general population of the city of São Paulo [23] to 1.7% in an Amerindian population in the Brazilian Amazon region [24]. Other studies have been carried out involving specific groups, such as blood donors (0.9%) [25], intravenous drug users (69.0%) [26], dialysis patients (23.8%-52.0%) [27-29], HIV patients (17.7%) [30], prisoners (16.0%) [31] and ex-soccer players (7.5%), who are considered to be at high risk of HCV infection associated with the intravenous injections of vitamins and the use of stimulants before games [32]. As there is no vaccine against HCV, the identification of infected individuals is mandatory for preventing the increasing prevalence of the disease [9,20].

Although the WHO states that dentists are at greater risk of HCV, a number of studies have shown that the prevalence of HCV infection in this group is similar (1.2%) [33] or even lower (0.0%) [34] than that of the general population [9]. The possibility of becoming infected by HCV is most commonly related to age and work experience [35]. Two studies carried out in mid-sized cities throughout Brazil describe a prevalence of 0.7% [35] and 0.4% [36] among dentists. However, there are no studies on the prevalence of HCV among dentists in large urban centres in Brazil.

Thus, the aim of the present study was to investigate the seroprevalence and sociodemographic data, health-related and occupational factors and other correlates of sero-posivity among dentists in the city of Belo Horizonte, MG, Brazil.

## Methods

### Sample/Population description

Belo Horizonte is the capital of the state of Minas Gerais (Brazil). It is an industrialized city with about 2.4 million inhabitants and considerable social, economic and cultural disparities. There are 2766 dentists registered at the Minas Gerais Dental Council and working regularly in Belo Horizonte [37]. These dentists were first contacted, enrolled and invited to take part in this study in November 2004 when all dentists registered at the Minas Gerais Dental Council were required to elect the administrative board of the council. A day was scheduled at a clinical analysis laboratory at the Medical School of the Federal University of Minas Gerais for the dentists who chose to take part in the study. We also published notes on the risks of hepatitis C in dental practice and the importance of undergoing the test in the Dental Council newsletter, the goal of which was to invite dentists to take part in the study.

The sample size was calculated to give a 95% CI, a level of precision of 0.75% [37] and using 3% prevalence of hepatitis C [9]. The inclusion criteria were dentists, who lived and worked in the city of Belo Horizonte and registered at the Minas Gerais Dental Council. This group consists of a finite population of 2766 dentists. The minimum sample size to satisfy the requirements was estimated at 1156 dentists. Taking into account the possibility of losses of previously enrolled dentists, a correction factor of 1.2 was adopted, totalizing an expected sample size of 1387 dentists. These dentists (n = 1387) scheduled a visit at the clinical analysis laboratory at the Medical School of the Federal University of Minas Gerais. In sequence, a total sample of 1302 dentists (response rate = 93.9%) answered the questionnaire and had a blood sample collected between December 2004 and June 2006. All participants had signed a term of informed consent and data were collected on demographic, behavioural and occupational exposure aspects.

### Questionnaire

A self-administered questionnaire consisting of twenty open-ended and close-ended items was used for the data collection. The drafting of this questionnaire complied with all steps proposed by previous studies [38,39]. Once the purpose of the study and its conceptual basis were defined, the drafting of the items was carried out by means of a broad-based review of the literature [3,7,33,40]. Content validation was performed to determine the suitability of the theoretical content and functionality of the questionnaire. Item selection, adaptation and additional inclusions were then performed based on the opinion of an expert in research and marketing. Subsequently, an opinion was formulated by a commission made up of professionals from different dental institutions and specialties. The commission members and the expert were aware of the objectives and methodology of the study and were asked to express their opinions on the writing and understanding of the questions. Unanimity in the approval of the questionnaire was required for validation. Suggestions for changes were heeded when brought up repeatedly by different commission members. Response options were organized vertically. All survey items were constructed in the same format in order to avoid placing emphasis on any specific item. Space was included for participants to answer the question when the multiple choice form was not possible.

The following variables were taken into account:

#### 1- Personal and behavioural data

Gender; sexual behaviour (unprotected homo/hetero sex with a casual partner); blood transfusions; knowledge of the results of hepatitis A and C tests; liver pain; abdominal pain; previous history of hepatitis in participant or family member; and the use of illicit injection drug. Since the latter variable is quite sensitive, this point was addressed in combination with exposure factors, such as the use of piercing and tattoos, history of any kind of transplant, dialysis, colonoscopy and chemotherapy.

#### 2- Professional, behavioural and occupational exposure data

Work experience; workplace; field of work; compliance with Individual Protective Equipment (IPE); vaccination for hepatitis B; immunized against hepatitis B; history and number of needle stick accidents with visible bleeding; dental assistance for patients with hepatitis; and whether the dentists' clinical dental chart contains a question on a history of hepatitis.

As people may go to school at any time of their lives and the number of years of professional activity could be more important than age in representing their exposure to risk factors, the age of the participants was not inquired, but rather the duration of activity (work experience).

For the purpose of this study, the number of needle stick accidents was categorized into four groups: none; less than 5; 5 to 10; or more than 10 accidents. In order to analyze the influence of work experience, the sample was categorized into four groups: less than 10 years; 10 to 20 years; 21 to 30 years; and 30 years or more. The workplace was considered as public, private or both.

### Blood collection and laboratory tests

Blood samples were collected by peripheral venipuncture from all 1302 dentists. After centrifugation, all sera samples were frozen at -80°C until two hours prior to the test. The samples were tested for antibodies against hepatitis C (anti-HCV) using a commercially available enzyme-linked immunosorbent assay (ELISA) (ABBOTT/MUREX™, Anti-HCV 3.0, Chicago, IL, USA). The assay was calibrated to international standards, following the manufacturer's recommendations. Specificity is greater than 99% [41,42] and its sensibility is 97.2% [43].

So, individuals positive for anti-HCV were recalled for further evaluation at a later date. A detailed clinical examination was performed and blood samples were drawn to repeat the anti-HCV tests, serum ALT estimations, HCV RNA determinations and ultrasound liver examinations. The presence of HCV RNA in the anti-HCV-positive samples was assessed by reverse transcription-polymerase chain reaction (RT-PCR). RT-PCR is considered gold standard, with greater sensibility than other quantitative methods and specificity of 98% to 99% [41,42].

The Human Research Ethics Committee of the Federal University of Minas Gerais (Brazil) approved the protocol for this study. All voluntaries signed an informed consent form, where they were informed about the implications of the results of the tests. Furthermore, they were informed by the consent form about the implications of the results of the tests. Each participant received the results of the tests and information about their clinical implications.

Data were analyzed using the Statistical Package for the Social Sciences (SPSS for Windows, version 17.0, SPSS Inc, Chicago, IL, USA). Bivariate analysis was the initial analytic strategies (Fisher exact test). Afterwards, we performed multivariate logistic regression. The model adjustment was verified by Hosmer & Lemeshow test. Seroprevalence of hepatitis C was dependent variable. The significance level was set at α = 0.05.

## Results

A total of 68.4% of the dentists were female. Regarding specialty or field of work, 40.1% reported being general dentists and the others were distributed among the different specialties recognized by the Brazilian Federal Council of Dentistry.

Table 1 displays the distribution of seroprevalence and determinant factors of hepatitis C among Brazilian dentists. The seroprevalence of anti-HCV was 0.9% (95% IC 0.5-1.7%). The factors associated to the prevalence of hepatitis C were a history of blood transfusion (p = 0.002) and having undergone a test for hepatitis C (p = 0.015). Among the 11 dentists who were positive for HCV, 36.4% reported a history of blood transfusion and 27.3% reported having undergone a test of hepatitis C. Among the 1235 individuals who not positive for HCV, 5.0% reported a history of blood transfusion. Among the 1137 individuals negative for HCV, 4.8% reported having undergone the test. None of the other factors studied was associated with the seroprevalence of hepatitis C (p ≥ 0.05).

**Table 1:** Factors and seroprevalence of hepatitis C among Brazilian dentists, Belo Horizonte, 2005

	Hepatitis C			p-value*
	**Present** **(%)**	**Absent** **(%)**	**Total** **(%)**	

Gender				
Male	3 (25.0)	408 (31.6)	411 (31.6)	0.762
Female	9 (75.0)	882 (68.4)	891 (68.4)	
Total	12 (100.0)	1290 (100.0)	1302 (100.0)	

Work experience†				
Less than 10 years	2 (16.7)	349 (27.6)	351 (27.5)	0.233
10 - 20 years	5 (41.6)	339 (26.7)	344 (26.9)	
21 - 30 years	2 (16.7)	420 (33.2)	422 (33.0)	
More than 30 years	3 (25.0)	158 (12.5)	161 (12.6)	
Total	12 (100.0)	1266 (100.0)	1278 (100.0)	

Workplace†				
Public	1 (9.1)	276 (23.3)	277 (23.2)	0.301
Private	4 (36.4)	536 (45.3)	540 (45.2)	
Both	6 (54.5)	372 (31.4)	378 (31.6)	
Total	11 (100.0)	1184 (100.0)	1195 (100.0)	
Field of Work†				
General dentistry	6 (66.7)	514 (47.6)	520 (47.7)	0.111
Oral Surgery/Periodontology	0 (0.0)	159 (14.7)	159 (14.6)	
Operative Dentistry/Endodontics	0 (0.0)	183 (16.9)	183 (16.8)	
Paediatric Dentistry/Orthodontis	1 (11.1)	157 (14.5)	158 (14.5)	
Oral Public Health	1 (11.1)	44 (4.1)	45 (4.1)	
Oral Pathology/Oral Radiology	1 (11.1)	24 (2.2)	25 (2.3)	
Total	9 (100.0)	1081 (100.0)	1090 (100.0)	

IPE use				
Yes	7 (58.3)	842 (65.3)	849 (65.2)	0.762
No	5 (41.7)	448 (34.7)	453 (34.8)	
Total	12 (100.0)	1290 (100.0)	1302 (100.0)	
Vaccinated for Hepatitis B†				
Yes	9 (90.0)	953 (84.8)	962 (84.8)	1.000
No	1 (10.0)	171 (15.2)	172 (15.2)	
Total	10 (100.0)	1124 (100.0)	1134 (100.0)	

Immunized against Hepatitis B†				
Yes	2 (22.2)	191 (19.8)	193 (19.8)	1.000
No	7 (77.8)	774 (80.2)	781 (80.2)	
Total	9 (100.0)	965 (100.0)	974 (100.0)	

Needle stick accidents†				
Yes	12 (100.0)	1091 (85.1)	1103 (85.2)	0.233
No	0 (0.0)	191 (14.9)	191 (14.8)	
Total	12 (100.0)	1282 (100.0)	1294 (100.0)	

Number of needle stick accidents†				
None	0 (0.0)	191 (15.0)	191 (14.8)	0.495
Less than 5	8 (66.6)	651 (51.0)	659 (51.2)	
5 - 10	2 (16.7)	264 (20.7)	266 (20.7)	
More than 10	2 (16.7)	170 (13.3)	172 (13.3)	
Total	12 (100.0)	1276 (100.0)	1288 (100.0)	

Hepatitis patient dental assistance†				
Yes	2 (20.0)	362 (35.9)	364 (35.7)	0.345
No	8 (80.0)	637 (63.1)	645 (63.3)	
Total	10 (100.0)	1009 (100.0)	1019 (100.0)	

Dental history question about hepatitis†				
Yes	11 (100.0)	1063 (83.3)	1074 (83.4)	0.228
No	0 (0.0)	213 (16.7)	213 (16.6)	
Total	11 (100.0)	1276 (100.0)	1287 (100.0)	

Unprotected homo/hetero sex†				
Yes	3 (25.0)	264 (20.6)	267 (20.7)	0.721
No	9 (75.0)	1015 (79.4)	1024 (79.3)	
Total	12 (100.0)	1279 (100.0)	1291 (100.0)	

Blood transfusion†				
Yes	4 (36.4)	62 (5.0)	66 (5.3)	0.002
No	7 (63.6)	1173 (95.0)	1180 (94.7)	
Total	11 (100.0)	1235 (100.0)	1246 (100.0)	

Knowledge of hepatitis A test result†				
Yes	2 (18.2)	58 (5.3)	60 (5.4)	0.115
No	9 (81.8)	1042 (94.7)	1051 (94.6)	
Total	11 (100.0)	1100 (100.0)	1111 (100.0)	

Knowledge of hepatitis C test result†				
Yes	3 (27.3)	55 (4.8)	58 (5.1)	0.015
No	8 (72.8)	1082 (95.2)	1090 (94.9)	
Total	11 (100.0)	1137 (100.0)	1148 (100.0)	

Liver pain†				
Yes	1	66 (5.5)	67 (5.3)	0.478
No	11	1197 (94.5)	1208 (94.7)	
Total	12 (100.0)	1208 (100.0)	1275 (100.0)	

Abdominal pain†				
Yes	0	98 (7.7)	98 (7.6)	1.000
No	12	1177 (92.3)	1189 (92.4)	
Total	12 (100.0)	1275 (100.0)	1287 (100.0)	

Previous history of hepatitis†				
Yes	2	129 (10.5)	131 (10.6)	0.369
No	10	1094 (89.5)	1104 (89.4)	
Total	12 (100.0)	1223 (100.0)	1235 (100.0)	

History of hepatitis in family†				
Yes	5	291 (29.3)	296 (29.5)	0.171
No	5	702 (70.7)	707 (70.5)	
Total	10 (100.0)	993 (100.0)	1003 (100.0)	

Combined question†, ††				
Yes	1	163 (13.1)	164 (13.0)	1.000
No	11	1082 (86.9)	1093 (87.0)	
Total	12 (100.0)	1245 (100.0)	1257 (100.0)	

* Fisher's exact test

† For these variables, the sum of the data does not result in 100%, as these questions were not answered by the entire sample.

††: This question included use of illicit injection drugs, piercings and tattoos, blood transfusion, transplant, history of dialysis, colonoscopy or chemotherapy.

Considering that two independent variables were associated to seroprevalence, we performed multivariate logistic regression. However the model did not converge. Thus, Fisher exact test was performed to evaluate the association between these two variables ("Test for hepatitis C" and "Blood transfusion"). Results of this test (p = 0.523) showed that there was no association between these variables and indicated that both were independently associated to seroprevalence (Table 2).

**Table 2:** Association between having undergone test of hepatitis C and history of blood transfusion, Belo Horizonte, 2005

		Blood transfusion			p-value*
			
		Yes (%)	No (%)	Total (%)	
Test for hepatitis C	Yes	4 (7.3)	53 (5.0)	57 (5.2)	0.523
	No	51 (92.7)	998 (95.0)	1049 (94.8)	
	Total	55 (100.0)	1051 (100.0)	1106 (100.0)	

* Fisher's exact test

## Discussion

The prevalence of chronic HCV infection in the general population is not uniform across countries and ranges from 0.1 to 5%, with an extraordinary high prevalence reported in Egypt (20-25%) [11]. The transmission of blood-borne viruses in dental offices is a potential hazard to patients and dental staff, particularly to oral and maxillofacial surgeons [5]. Thus, the present study focused on the prevalence of anti-HCV among dentists in the city of Belo Horizonte, Brazil. The overall seroprevalence of HCV was 0.9% among a calculated sample of 1302 dentists tested. This is lower than the 1.42% prevalence of HCV reported for the general population [23] and 1.7% among Amazon Amerindian populations [24]. However, the prevalence of hepatitis C among dentists reported here is higher than the 0.0095% found in a recent similar study [43] involving 1056 Swiss dental healthcare workers (834 dentists; 222 dental hygienists and assistants). The prevalence is also higher than that identified in mid-size Brazilian cities, such as Sertãozinho [35] and Piracicaba [36]. It is important to notice that none of these Brazilian studies had a randomized sample. Despite the sample of the present study is not a randomized sample, our sample is the biggest sample among these Brazilian studies. Moreover, data provided by Minas Gerais Dental Council showed that the proportion of female dentists was of 62% among the population of dentists in Belo Horizonte. This data was very similar to our sample (68% of female dentists).

The low prevalence of HCV in dentists described in a previous report [44] and confirmed here might suggest that dentists have a lower risk of HCV infection caused by occupational accidents than expected by the WHO [9]. Professional data, behaviour and occupational exposure were not associated to the seroprevalence of hepatitis C. This may be explained by the relatively low infectivity of HCV found in saliva or by the small volume of blood inoculation involved in dental accidents [44,45]. The participants' responses were self-reported; hence, caution with generalizations must be considered. Volunteers may have habits or behaviour different from their self-reported behaviour, meaning that some level of information bias could exist.

In our study all dentists with positive results were considered asymptomatic and only three knew of their condition as an HCV carrier. These dentists were offered further medical assistance by hepatologists. Thus, the investigation gave them the opportunity to become aware of their status as a HCV carrier. There are several advantages to the early diagnosis of hepatitis C. Infected individuals must be made aware of self-care and behaviour that could hasten the progression of liver disease, such as alcohol intake. In addition, early diagnosis allows infected people to receive appropriate preventive services (e.g., hepatitis A and B vaccination) and to be referred to a clinic for evaluation and HCV therapy [11,21].

The association found between blood transfusion and hepatitis C reveals dentists have a predisposing factor to this disease that is common to the general populations. The risk of contamination with hepatitis C through a blood transfusion is currently very low in the world [46,47]. Prior to 1992, however, no HCV detection test was carried out on blood donors [19]. In Brazil, tests for the detection of HCV among blood donors were only mandatory beginning in 1993.

Although the prevalence of hepatitis C found in this study was low, the number of reported accidents with needle sticks was unexpectedly high, suggesting that permanent vigilance and educational programs addressing hepatitis C virus infection among dentists are necessary. Although the probability of infection due to contaminated needle sticks is lower for HCV than HBV [34], this is an important issue considering the seroprevalence of hepatitis C in the Brazilian population [23-32] and the issue of dentists being exposed often to this type of accident [34].

The present study has some limitations that must be recognized. Cross-sectional studies are carried out either at a single point in time or over a short period. Thus, associations identified in cross-sectional studies should not be considered a causal relationship. However, there is a lack of studies that concomitantly assess different variables associated to hepatitis C among dentists, as we performed in the present study.

As no vaccine or immunoglobulin HCIg against HCV is available, it is virtually impossible to prevent HCV infection after occupational exposure. Thus, every precaution must be taken to prevent infection, including the screening and testing of blood and organ donors; the inactivation of the HCV virus in plasma-derived products, the permanent vigilance and maintenance of the best preventive practices in healthcare settings, including the need for continual infection control policies in dentistry and the proper treatment of contaminated patients [9,21].

## Conclusions

The seroprevalence of anti-HCV among dentists was low and no occupational exposure conditions were associated to the seroprevalence of hepatitis C.

## Competing interests

The authors declare that they have no competing interests.

## Authors' contributions

VLSR, MHGA, SMP, RT and IAP conceptualized the rationale and design of the study. VLSR and MHGA performed the statistical analysis and interpretation of the data. VLSR, MHGA, SMP and IAP drafted the manuscript. All authors read and approved the final manuscript.
